# Children’s psychiatric medical history. Review

**DOI:** 10.1192/j.eurpsy.2021.1667

**Published:** 2021-08-13

**Authors:** M.O. Solis, F. Vilchez Español, A. Alvarado Dafonte, M. ValverDe Barea, S. Jimenez Fernandez

**Affiliations:** 1 Jaén, Complejo Hospitalario Jaén, Jaén, Spain; 2 Mental Health Unit, Complejo Hospitalario de Jaen, Jaen, Spain

**Keywords:** medical history, CHILDREN’S PSYCHIATRIC, adolescents, psychopathology

## Abstract

**Introduction:**

The prevalence of mental disorders in children and adolescents varies between 5 and 22%, depending on the methodology, type of interview, samples and inclusion of the disability criterion. Between 4 and 6% of children and adolescents have severe mental disorders.

**Objectives:**

Reason for consultation. Current disease Milestones of psychomotor development. The presence of abnormal behaviors, delays in motor development, speech and socialization will be specified. As the child’s behavior depends to a large extent on the context, specific methods should be used to evaluate the child’s behavior at home, at school and in the clinical situation. Complementary exams: Genetic testing. Blood and urine tests, including toxics. EEG, polysomnography and evoked potentials. X-rays, CT-scans, MRI.

**Methods:**

The essential source of medical history is clinical interviews. The semi-structured format is the most recommended by the different authors, because it allows some flexibility in the realization of the story, while providing a baseline to develop the interview (J. Diaz Atienza).

**Results:**

The diagnostic formulation must be individualized without assigning a categorial psychiatric diagnosis. (Doménech E et al).

**Conclusions:**

The main and irreplaceable evaluation technique remains the medical history. It is important to take into account the reason for consultation and the context of both the child’s family and its ethnic, cultural and ethical characteristics. It is of the utmost importance to have and evaluate the stages of normal development and to adapt to the age that our patient has.

**Disclosure:**

No significant relationships.

